# Challenging paradigms: long non-coding RNAs in breast ductal carcinoma *in situ* (DCIS)

**DOI:** 10.3389/fgene.2013.00050

**Published:** 2013-04-09

**Authors:** Mary A. Kosir, Hui Jia, Donghong Ju, Leonard Lipovich

**Affiliations:** ^1^Department of Surgery, Wayne State University School of MedicineDetroit, MI, USA; ^2^Department of Oncology, Karmanos Cancer Institute, Wayne State University School of MedicineDetroit, MI, USA; ^3^Center for Molecular Medicine and Genetics, and Department of Neurology, Wayne State University School of MedicineDetroit, MI, USA

## Overview

The continuing challenges to the treatment of breast ductal carcinoma *in situ* (DCIS) warrant a new avenue of investigation. In parallel, a startling revelation of the first post-genomic decade has been the rising prominence of long non-coding RNAs (lncRNAs) as key gene regulators, including in breast cancer. High-throughput experimental surveys of the human transcriptome show that lncRNAs are abundant. In contrast to microRNAs (miRNAs), lncRNAs possess multiple, diverse, heterogeneous molecular mechanisms of action making them high-level master regulators of gene regulatory networks and pathways in cancer. In particular, lncRNAs directly regulate the activity of transcription proteins and epigenetic modifiers, which, in turn, govern their own downstream sets of target genes. Therefore, provided that such lncRNA regulatory activities are functional in cancer, including DCIS, targeting the lncRNAs or the regulatory networks which they mediate can represent an important new avenue of treatment. Development of lncRNA-targeting therapies may emerge into a key innovation generated in clinical and translational research by the innovative use of the latest human genome annotation datasets combined with high throughput transcriptome analyses. We propose a paradigm shift: direct interrogation of lncRNAs in the molecular characterization of DCIS subtypes, including the idea that lncRNAs may be the trigger for progression from *in situ* to invasive cancer.

## Challenges of DCIS

The National Institutes of Health recommendations for research on DCIS (Allegra et al., 2010) remain active at this time. The challenges that make DCIS intractable to treatment have been the heterogeneity of DCIS and its shared expression of molecules with invasive breast cancer. While the phenotype is preinvasive, the proliferative, angiogenenic, and even invasive characteristics of DCIS are shared with its invasive counterpart. Kretschmer et al. (2011) have identified overexpressed genes in both DCIS and invasive breast cancer. More recently, non-coding RNA has become a focus of interest as this might organize (or disorganize) the expression of molecules for the DCIS phenotype. Hannofon et al. (2011) evaluated miRNAs expressed in the change from “normal” to preinvasive DCIS. However, there are two reasons for the importance of characterizing and functionally interrogating lncRNAs in DCIS. miRNAs are primarily post-transcriptional suppressors, but lncRNAs allow us to access a diversity of epigenetic, co-transcriptional, and post-transcriptional mechanisms of gene regulation, which are not used by miRNAs. Secondly, lncRNAs themselves represent direct functional targets, while miRNAs are not because each microRNA is a post-transcriptional suppressor of hundreds or thousands of potential distinct gene targets. So miRNAs are not necessarily useful themselves as direct targets for therapies, although they may lead to mRNAs of target genes. LncRNAs are frequently involved in regulatory modalities, such as unique protein–RNA interactions in ribonucleoprotein complexes (RNP), or sense–antisense RNA regulation, which are highly specific and therefore lack counterparts in the microRNA world. Because lncRNAs are high-level regulators of gene expression in cancer, targeting lncRNAs may likely allow therapeutics directed toward a cause, rather than toward a consequence, of the regulatory networks operant in DCIS (Lipovich et al., 2010). There is current and rapidly growing evidence supporting distinct lncRNA roles in breast cancer, and existing public gene expression datasets, including DCIS microarray data and RNASequencing (RNAseq), can be interrogated for lncRNA differential expression.

## Current state of the field

Computational integration of the human genome with experimentally determined transcript sequences has revealed that half of human genes encode non-protein-coding RNAs (ncRNAs): miRNAs and the more numerous lncRNA transcripts. Key technological advances have led to large-scale cloning and sequencing of complementary DNAs (cDNAs), which are derived by reverse transcription from mature, full-length, 5′-capped, polyAtailed RNAs, thereby validating, and inferring new gene structures empirically from cDNA to genome alignments, and to evaluate each experimentally documented transcript for protein-coding potential. This method was used by the Functional Annotation of Mouse (FANTOM) project to show that non-coding transcription is widespread, and that the number of lncRNA transcriptional units in the mammalian genome approximately equals that of protein-coding genes (Carninci et al., 2005; Carninci and Hayashizaki, 2007). Next Generation Sequencing (NGS) technologies, including RNAseq, have further increased the number of known lncRNAs (Cabili et al., 2011).

LncRNAs exert key regulatory functions, and control many normal and disease processes. Knockdown and overexpression of specific lncRNAs generate reproducible phenotypes, because the lncRNAs regulate protein-coding genes, by multiple mechanisms (Lipovich et al., 2010). Many lncRNA mechanisms directly function in cancer. Specific known lncRNAs promote transformation as oncogene targets; are diagnostic markers and cell cycle regulators in cancer; mediate cell survival; regulate mRNA splicing; and directly regulate epigenetic and transcription factors (Lipovich et al., 2010). Gutschner and Diederichs (2012) have catalogued lncRNAs into cancer-specific activities which are relevant to DCIS such as sustained signaling for proliferation, immortality, angiogenesis, resisting cell death, avoiding suppressors. In more aggressive DCIS, there may also be activation of invasion and ultimately metastasis as DCIS progresses.

With a small number of lncRNAs with known functions, the abundance of lncRNA functions in the cancer literature is noteworthy. P53, perhaps the most famous tumor suppressor, is co-activated by MEG3, an imprinted lncRNA (Zhou et al., 2007), and in turn, regulates an additional lncRNA as a global effector of gene expression changes (Huarte et al., 2010). Another imprinted lncRNA, H19, is a direct target of C-Myc and is functional in cell proliferation (Barsyte-Lovejoy et al., 2006). Sense–antisense mRNAlncRNA gene pairs affect several tumor suppressors which are cis-regulated by antisense lncRNAs transcribed from the same locus, including p15 and p21 (Lipovich et al., 2010). A prostate-specific lncRNA, PCGEM1, is associated with prostate cancer and regulates apoptosis (Fu et al., 2006), while RNAseq has uncovered a second lncRNA in prostate cancer progression (Prensner et al., 2011). Highly Upregulated in Liver Cancer (HULC), identified as the most-upregulated gene in an HCC microarray study of 7000 genes, is a canonically spliced, polyadenylated lncRNA whose knockdown in a cell line induces CDK8 and a tumor suppressor candidate (Panzitt et al., 2007). The nuclear lncRNAs NEAT1 and NEAT2, resident in and essential for nuclear paraspeckles and speckles respectively, have diverse cancer functions as well. NEAT2 (MALAT-1) is a marker of diverse human carcinomas, and a predictor of survival and metastasis across multiple cancer types (Lin et al., 2007). Telomerase, a RNP with crucial functions in cancer, which contains a highly conserved lncRNA (Blackburn, 2005), became the subject of a Nobel Prize in 2009. Additional lncRNAs functional in proliferation and with anti-apoptotic roles are still being discovered (Hu et al., 2011), while other lncRNAs which have not yet been functionally validated are emerging as highly significant cancer biomarkers (Gloss et al., 2012).

There is increasing evidence for lncRNA function in human breast cancer. Specific lncRNAs are predictors of metastasis and survival; directly function in cell proliferation and cell cycle in breast cancer; and directly regulate nuclear hormone receptor function in breast cancer (Gupta et al., 2010; Lipovich et al., 2010; Silva et al., 2011). LSINCT5 is a polyadenylated lncRNA expressed at greater levels in breast and ovarian tumor tissues and cell lines relative to normal tissues. Knockdown of LSINCT5 decreases cellular proliferation (Silva et al., 2011). HOTAIR, an lncRNA whose expression is increased in primary breast tumors and their metastases, induces genome wide retargeting of the Polycomb repressor complex 2 (PRC2), which causes global gene expression changes that facilitate invasiveness and metastasis (Gupta et al., 2010). The lncRNA PINC, expressed in the regressed terminal ductal lobular unit-like structures of the mammary gland, is functional in cell survival and cell cycle progression (Ginger et al., 2006). The bifunctional lncRNA SRA is a co-activator of estrogen receptor alpha (Lipovich et al., 2010). Zfas1, another bifunctional lncRNA, which can serve as a snoRNA host transcript or as a stand alone lncRNA molecule, localizes to the ducts and alveoli of the mammary gland, and has an expression profile independent of the encoded snoRNAs, supporting its distinct role. Zfas1 is downregulated in breast tumors, potentially due to a tumor suppressor function (Askarian-Amiri et al., 2011).

## Perspective on future research for DCIS

Despite the wealth of recent evidence for lncRNA function in cancer, including breast cancer, no studies to date have pinpointed lncRNAs as functional determinants, biomarkers, or treatment targets in DCIS. A strategy to assess lncRNA differential expression in DCIS samples to identify functional lncRNAs includes the use of existing (catalog) microarray designs, custom microarrays, and RNAseq. Although RNAseq is unbiased, while custom microarrays can maximize the coverage of poorly-annotated lncRNAs, most DCIS gene expression profiling to date has used conventional microarray designs from established microarray manufacturers. We have recently performed genome wide identification of lncRNA genes from public ncRNA datasets and full-length cDNA data (Jia et al., 2010). To determine the extent to which existing commercial microarray platforms, such as the still widely used Affymetrix U133A and B chips, can be employed for lncRNA differential expression discovery, we performed genomic positional overlaps between commonly used commercial microarray probesets and our over 6700 lncRNAs. We show that 43% of our lncRNAs have some U133 probeset representation, and list these lncRNAs in Supplemental Data Set 4 (Jia et al., 2010). In another disease system, we have validated the paradigm of interrogating existing Affymetrix U133 datasets to identify lncRNA differential expression (Michelhaugh et al., 2011). Here, we propose to apply the same paradigm to discover DCIS-specific lncRNA differential expression. We have previously used our annotation approach (which relates public microarray probes to genes which we know to be lncRNAs) to identify putative functional lncRNAs. Data re-mining of existing public cancer array datasets can easily identify lncRNA genes associated with the phenotypes or regulatory systems for DCIS. Depending on the sample properties and the experimental design of the DCIS microarray experiments whose results are in the public domain, specific lncRNAs can be identified directly from the public datasets, by determining which differentially expressed unannotated probesets in the public data reflect lncRNAs. These lncRNAs can be further validated by qRTPCR to confirm their differential expression in other samples or in cell line models, and they can then be interrogated by reverse genetic experiments in applicable cell line models (system perturbations: RNAi and overexpression) to elucidate any potential causal relationship to a mechanism or phenotype. Such validation of these lncRNAs as contributors to the pathogenesis of DCIS is a prerequisite for declaring them to be putative drug targets.

We have identified at least four studies which generated Affymetrix U133 microarray public data repositories from clinical DCIS samples (Schuetz et al., 2006; Turashvili et al., 2007; Hawthorn et al., 2010; Gabrovska et al., 2011). These studies included lncRNAs because 43% are represented by unannotated, accession-number-only, and “hypothetical-protein” gene probesets on the microarray. Schuetz et al. (2006) focused on differentially expressed genes marking the transition of stationary epithelial cells to migrating invasive cells, and used patient-matched DCIS/invasive ductal carcinoma (IDC) samples. Gabrovska et al. (2011) aimed to develop grade-specific gene expression signatures from paraffin-embedded IDC tissue samples, including benign tumors, and identified a larger set of unannotated gene signature members which can be mined for lncRNAs. Turashvili et al. (2007) identified genes whose expression differed between IDC and invasive lobular carcinoma, as well as between IDC and normal cells. Hawthorn et al. (2010) simultaneously examined genomic copy number variation and gene expression in IDC, highlighting genes whose transcript levels change as a result of genomic deletions.

## Conclusions

There is great potential in studying non-coding RNA in DCIS. In a paradigm-shifting approach, lncRNAs that are currently contained in published data sets, in particular the unannotated lncRNAs represented by Affymetrix probesets, are available to investigate DCIS. To the extent that the corresponding clinical datasets are represented by public microarray repositories, these lncRNAs can be utilized to further characterize the heterogeneous nature of DCIS, and to elucidate the mechanisms that support preinvasive DCIS and the progression to invasive breast cancer. The pace of future functional DCIS lncRNA discovery can be substantially accelerated if unbiased whole-transcriptome differential expression profiling via RNAseq, further aided by including the diverse public lncRNA datasets such as those from ENCODE and the Broad Institute, becomes a reality in the analysis of DCIS clinical samples.
